# SA-UMamba: Spatial attention convolutional neural networks for medical image segmentation

**DOI:** 10.1371/journal.pone.0325899

**Published:** 2025-06-12

**Authors:** Lei Liu, Zhao Huang, Shuai Wang, Jun Wang, Baosen Liu

**Affiliations:** 1 School of Computer Science and Technology, Huaibei Normal University, Huaibei, Anhui, China; 2 Huaibei Key Laboratory of Digital Multimedia Intelligent Information Processing, Huaibei, Anhui, China; 3 College of Electronic and Information Engineering, Hebei University, Baoding, Hebei, China; 4 Huaibei People’s Hospital, Huaibei, Anhui, China; Khalifa University of Science and Technology, UNITED ARAB EMIRATES

## Abstract

Medical image segmentation plays an important role in medical diagnosis and treatment. Most recent medical image segmentation methods are based on a convolutional neural network (CNN) or Transformer model. However, CNN-based methods are limited by locality, whereas Transformer-based methods are constrained by the quadratic complexity of attention computations. Alternatively, the state-space model-based Mamba architecture has garnered widespread attention owing to its linear computational complexity for global modeling. However, Mamba and its variants are still limited in their ability to extract local receptive field features. To address this limitation, we propose a novel residual spatial state-space (RSSS) block that enhances spatial feature extraction by integrating global and local representations. The RSSS block combines the Mamba module for capturing global dependencies with a receptive field attention convolution (RFAC) module to extract location-sensitive local patterns. Furthermore, we introduce a residual adjust strategy to dynamically fuse global and local information, improving spatial expressiveness. Based on the RSSS block, we design a U-shaped SA-UMamba segmentation framework that effectively captures multi-scale spatial context across different stages. Experiments conducted on the Synapse, ISIC17, ISIC18 and CVC-ClinicDB datasets validate the segmentation performance of our proposed SA-UMamba framework.

## Introduction

Medical segmentation plays a crucial role in modern clinical applications, such as assisting in diagnosis, helping to formulate treatment plans, and guiding the implementation of treatment methods [1–3]. In practice, the analysis of medical segmentation results relies on experienced doctors. However, owing to the subjective and objective differences in doctors’ judgments [4], it is impossible to rapidly obtain judgments that are also accurate, leading to discrepancies between reality and the results of analysis. Therefore, accurate and fast medical image segmentation methods are very important, as they can not only enhance diagnostic efficiency [5], but also ensure the accuracy of the results.

In recent years, convolutional neural network (CNN)- and Transformer-based deep learning methods [6, 6–9] have significantly contributed to advance the field of medical image segmentation. Among them, U-Net [6], as a representative CNN method, has been applied in subsequent medical image segmentation studies because of its simple framework. Many subsequent studies [11–14] have adopted this U-shaped structure and achieved promising results. However, CNN-based methods [7, 8, 11] are limited by their local receptive fields, which prevent them from effectively modeling long-range dependencies. Inspired by the success of Transformer-based methods [15–17] on natural language processing (NLP) and image tasks, TransUNet [9] was the first to apply a Vision Transformer (ViT) to medical image segmentation, demonstrating the powerful performance of Transformers in this domain. Subsequently, TransFuse [18] was designed with a ViT [16] in the encoder stage and CNN in the decoder stage, capturing both global and local features simultaneously. Additionally, Swin-Unet [10] employs the Swin Transformer [17] in both the encoder and decoder stages; it was demonstrated to outperform pure convolutional and hybrid Transformer-based methods. Although Transformer-based methods excel in global modeling, their unique self-attention mechanism introduces quadratic computational complexity. Particularly in medical image segmentation, as image resolution and data volume increase, the computational requirements of the model significantly increase, thereby creating a substantial computational burden [16, 19, 20]. Therefore, there is an urgent need for a new medical image segmentation architecture that can effectively capture long-range dependencies while maintaining linear computational complexity, thus mitigating the rising computational cost.

Recently, state-space models (SSMs) [21, 22] have demonstrated their effectiveness in long-sequence modeling. Compared to Transformers, they have linear computational complexity, scaling linearly with the sequence length. Mamba [23] emerged as a result of applying a SSM to end-to-end neural networks, retaining the abilities of SSM while significantly enhancing the inference capability beyond that feasible for Transformers. Owing to Mamba’s success in continuous long data analysis in fields such as NLP and genomic analysis, researchers have recently begun exploring its application in the visual domain [24–31], consequently achieving successful outcomes. Vision Mamba [24] was constructed a Mamba framework similar to the ViT structure, being the first to apply Mamba in the visual domain. Subsequently, the VMamba [25] architecture introduced the 2D selective scanning (SS2D) module, which enables image patch scanning in four directions and thus enhances Mamba’s ability to understand spatial information in images. Mamba was then integrated into U-shaped medical image segmentation architectures. Specifically, U-Mamba [26] was the first to apply the original Mamba module to medical image segmentation, whereas VM-UNet [28] and Swin-UMamba [29] leveraged the Swin-Unet architecture and VMamba’s pre-trained weights, outperforming methods like Swin-Unet on medical segmentation datasets and thus showcasing strong competitiveness. Recently, some Mamba-based methods [29, 30] have incorporated spatial and channel attention; however, they focus on global spatial or channel attention rather than concentrating on spatial attention within the receptive field, which limits their ability to effectively capture fine-grained spatial features in the local context. Furthermore, co-optimization of the feature extraction capability of Mamba and spatial attention remains a challenge.

To comprehensively address the limitations of existing methods in balancing global context and local spatial detail, we propose a novel residual spatial state-space (RSSS) block as an extension of the VSS block from VMamba. The RSSS block integrates a receptive-field attention convolution (RFAC) module to enhance spatial awareness by capturing position-specific features within the receptive field. We have also incorporated learnable parameters, positioning them at both ends of the VSS block and spatial module to act as adjustment factors for the residual connection and co-optimize the Mamba-derived features and spatial attention. We have also constructed the RSSS block to have an architecture that is similar to VM-UNet, named SA-UMamba, which enhances segmentation performance. Briefly, the contributions of this paper are as follows:

- We propose a RSSS block, which efficiently integrates Mamba with RFAC module to simultaneously capture global dependencies and spatially diverse local features;
- We have introduced learnable parameters to optimize the residual connections within the RSSS block and more effectively balance the Mamba-derived features and spatial attention to realize more comprehensive feature capture;
- We build SA-UMamba based on the RSSS block, consequently improving the Dice similarity coefficient (DSC) performance on the Synapse dataset, with the DSC increasing from 80.50% to 82.54%, and Hausdorff distance (HD95) decreasing from 22.37 mm to 16.80 mm. This demonstrates the leading effectiveness of the Mamba method on Synapse, as well as its competitiveness with the latest Transformer-based methods.

## Related works

### U-like medical image segmentation

Most early works in medical image segmentation were CNN-based methods inspired by U-Net [6]. Many subsequent works [7, 32–35] also applied this architecture, such as ResUNet [7] and UNet++ [32]. These methods were also extended to the field of 3D medical image segmentation, employing models such as the 3D U-Net [36] and V-Net [37]. These works benefit from the strong feature extraction capability of convolution, but they are also limited by the inability of convolution to extract global features. Initially, Transformers were only applied for translation tasks in NLP [15]. However, researchers recently began successfully applying them to the visual domain [16, 17], offering a new approach to medical image segmentation. For example, TransUNet [9] combines Transformer and CNN models to aggregate global and local feature information. Swin-Unet [10] employs Transformer blocks to form a complete Transformer segmentation framework on the basis of the U-shaped structure, achieving excellent segmentation performance based on global context. However, the attention mechanism in Transformers involves a high level of computational complexity, which means that Transformer-based methods require expensive hardware resources. Recently, the emergence of Mamba, with its linear computational complexity, has attracted widespread interest from researchers. Thus, Mamba was quickly adopted in the field of medical image segmentation, and Mamba-based models [23, 38] like VM-UNet [28], VM-UNetV2 [30] and Swin-UMamba [29] have achieved excellent segmentation performance in the medical segmentation field, offering a promising solution to the computational challenges of Transformer-based methods. Despite Mamba’s promising potential, its current applications remain limited, particularly in fully leveraging spatial attention mechanisms within the receptive field. Our work addresses this gap by introducing an optimized approach to integrate spatial attention within the receptive field, enhancing Mamba’s feature extraction capability for more precise segmentation.

### Attention mechanism

As an effective means of enhancing model performance, the attention mechanism allows models to focus more on key features. It is widely used in the fields of vision and NLP, establishing a relatively complete system. Wang *et al.* [39] proposed a convolutional block attention module (CBAM), which utilizes channel and spatial attention modules to capture both channel and spatial features. As a portable module, it not only enhances network performance, but also be easily applied to CNNs. Although the CBAM has already demonstrated good performance, Hou *et al.* [40] identified information loss within the CBAM and proposed the lightweight coordinate attention (CA) mechanism to address this problem. Zhang *et al.* [41] argued that previously developed attention mechanisms do not consider multi-scale feature information in channels and space. Thus, they proposed the PSA and SPC modules to enable multi-scale feature extraction in the channel and spatial dimensions, respectively. Although existing attention mechanisms can effectively enhance local feature representation, they often overlook the variations among different spatial positions within the local receptive field. Inspired by RFAC [42], we explored a new approach to spatial attention (referred to as spatial attention in this paper), using receptive-field spatial features to capture fine-grained positional variations, thereby further improving network performance. Additionally, to co-optimize spatial features and Mamba-derived features and thus enhance feature extraction capability, we introduced adjustment factors, which also serve to enhance the comprehensiveness of the model. Unlike most existing approaches [30, 45] that simply add attention blocks, our work establishes receptive-field spatial attention as an effective method that focuses on balancing these features, ensuring a more comprehensive and effective integration upon current research.

## Methods

**Fig 1:**
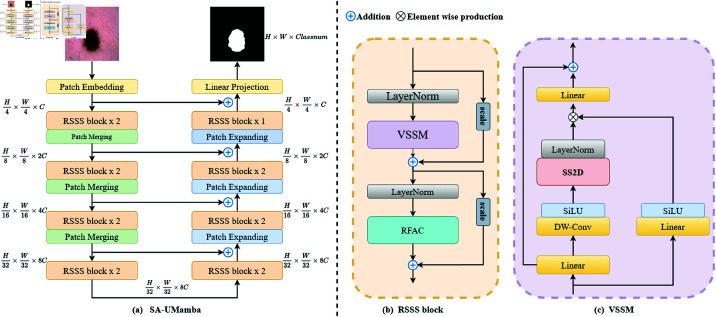
(a) The overall architecture of our proposed SA-UMamba. (b) RSSS block is the main construction block of SA-UMamba, integrating the VSSM and RFAC modules for enhanced feature extraction. (c) VSSM is the crucial module in the Mamba model for extracting visual features, with SS2D as its core operation.

In this section, we first introduce the concept of Mamba, and then describe our network structure in detail. Fig 1 shows the network structure of our proposed SA-UMamba, which employs an encoder-decoder architecture. Note that the encoder and decoder networks both comprise Mamba-based VSSS blocks.

### Mamba preliminaries

Current SSMs, namely, structured state space sequence models (S4) and Mamba, both rely on their continuous systems. These systems map 1D input functions or sequences (denoted as x(t)∈ℝ) to the output y(t)∈ℝ through an intermediate hidden state function $h(t)\in {\mathbb{R}}^{N}$. This process can be represented by a linear ordinary differential equation:

$$\left\{ \begin{array}{l} h^{'}(t)=Ah(t)+Bx(t),\\ y(t)=Ch(t)+Dx(t), \end{array}\right.$$ (1)

where $𝐀\in {\mathbb{R}}^{N\times N}$ represents the state matrix, $𝐁\in {\mathbb{R}}^{N\times 1}$ and $𝐂\in {\mathbb{R}}^{N\times 1}$ represent the linear projection parameters, and $𝐃\in {\mathbb{R}}^{1}$ represents the skip connection.

Mamba and S4 discretize the continuous system to better adapt to deep learning environments. They introduce the time scale parameter Δ and apply consistent discretization rules to convert **A** and **B** into the discrete parameters $\overset{―}{𝐀}$ and $\overset{―}{𝐁}$, respectively. The discretization method applies the zero-order hold approach, specifically represented as follows:

$$\left\{ \begin{array}{l} \bar{A}=\exp(\Delta A),\\ \bar{B}={\left(\Delta A\right)}^{-1}(\exp(\Delta A)-I)\cdot \Delta B. \end{array}\right.$$ (2)

After discretization, SSM-based models (S4 models) can be computed in two different ways: linear recursion or global convolution. These two computation methods are represented by Eqs 3 and 4, respectively, as shown below:

$$\left\{ \begin{array}{l} h^{'}(t)=\bar{A}h(t)+\bar{B}x(t),\\ y(t)=Ch(t)+Dx(t), \end{array}\right.$$ (3)

$$[10pt]\{\begin{array}{l} \bar{K}=(C\bar{B},C\bar{\text{AB}},...,C{\bar{A}}^{L-1}\bar{B}),\\ y=x*\bar{K}, \end{array}$$ (4)

where $\overset{―}{𝐊}\in {\mathbb{R}}^{L}$ represents the structured convolutional kernel, and *L* denotes the length of the input sequence *x*.

However, the SSM (S4) cannot make adjustments based on the input content, which significantly limits its ability to capture context and makes it difficult to achieve the same effect as Transformers. In contrast, the selective SSM, also referred to as Mamba or S6, improves upon this by introducing related parameters, i.e., *B* = *S*_*B*_(*x*), *C* = *S*_*c*_(*x*), and $\Delta =S_{\Delta }(x)$. This allows the model to adapt to more complex inputs and enable Mamba to maintain efficient training and inference through the scan algorithm.

### SA-UMamba framework

The overall architecture of SA-UMamba is shown in Fig 1 (a). SA-UMamba adopts an asymmetric design similar to VM-UNet [28]. Specifically, SA-UMamba comprises a patch embedding layer, an encoder, a decoder, a final projection layer, and skip connections, and the number of blocks in each encoder and decoder stage is determined based on VM-UNet.

First, the input image $x\in {\mathbb{R}}^{H\times W\times 3}$ is fed into the patch embedding layer. The patch embedding layer divides the image into non-overlapping 4 × 4 patches and applies a linear projection layer to expand the channels of each patch to *C*; it was initially set to 96. Through this process, the original image is reshaped into a feature map $x^{\prime }\in {\mathbb{R}}^{\frac{H}{4}\times \frac{W}{4}\times C}$, where *H* and *W* are the original height and width of the input image, respectively. Next, layer normalization [43] is applied to $x^{\prime }$, and the normalized $x^{\prime }$ is fed to the encoder for feature extraction.

The encoder comprises four stages, each using RSSS blocks to further extract features from the input. Additionally, in the first three stages, we apply patch-merging operations to process the input features, reducing the width and height by half while also doubling the number of channels. We apply [2,2,2,2] RSSS blocks in the four stages of the encoder, with the number of channels for each stage established as [*C*,2*C*,4*C*,8*C*].

The decoder also comprises four stages, similar to the encoder, using RSSS blocks to process the features. However, unlike the encoder, which processes features in the first three stages, the decoder uses patch-expanding operations in the last three stages to double the width and height of the feature maps while also halving the number of channels. In these four stages, we apply [2,2,2,1] RSSS blocks, with the number of channels for each stage established as [8*C*,4*C*,2*C*,*C*]. After being processed by the decoder and final linear layer, the width, height, and number of channels of the feature map are restored to the original image size, and this feature map is used to match the segmentation targets.

Standard residual connections are applied for the skip connections. Before the features are fed to the RSSS blocks of each encoder stage, skip connections are established at the corresponding positions in the decoder. These skip connections help to transmit information from the early stages of the encoder to the subsequent decoder stages, facilitating the network’s ability to retain and utilize these low-level features.

### Residual spatial state-space block

Previous Mamba-based medical image segmentation studies [28–30] generally adopted VSS blocks as the backbone for the encoder and decoder of the U-shaped network structure. The VSS block was designed based on VMamba’s VSS block [25]. Considering the results of various Transformer-based studies [44, 45], we believe that the features extracted by the VSS block can be further enhanced through additional spatial attention mechanisms. Therefore, a new structure specifically designed for Mamba-based medical image segmentation networks holds promise.

Thus, we propose the RSSS block as an enhancement of the Mamba-based VSS block. Specifically, we have applied RFAC as a new paradigm for spatial attention to enhance Mamba’s ability to extract spatial features. Our key idea is to facilitate feature co-optimization between global Mamba-derived spatial representations and local spatial attention and this is achieved by embedding residual connections with learnable adjustment factors, enabling adaptive information fusion.

As shown in Fig 1 (b), given the input feature $x\in {\mathbb{R}}^{H\times W\times C}$, we first apply LayerNorm for normalization, and then use the vision state-space module (VSSM) to capture long-term spatial dependencies. Additionally, to co-optimize the Mamba-derived spatial features and spatial attention features, we apply the learnable adjustment factor $s\in {\mathbb{R}}^{C}$ (denoted as "scale" in Fig 1 (b)) as a means to control the information from the residual connection. This operation allows the network to control the relative contribution of the original input to the enhanced representation and this process can be represented by the following equation:

$$Z^{l}=VSSM\left(LN\left(x\right)\right)+s\cdot x,$$ (5)

where *l* denotes the *l*-th RSSS block and *LN* represents the LayerNorm process. Subsequently, ${\text{Z}}^{\text{l}}$ is further normalized and fed into the RFAC module, which performs local spatial attention refinement. To integrate these spatially refined features with the global representation from VSSM, we introduce a second learnable scaling parameter $s^{\prime }\in {\mathbb{R}}^{C}$ in the residual connection, enabling dynamic co-optimization of global and local cues. This process can be represented by the following equation:

$$F_{D}^{l}=RFAC\left(LN\left(Z^{l}\right)\right)+s^{\prime }\cdot Z^{l}.$$ (6)

Through this two-stage integration strategy, the RSSS block explicitly models and balances the interaction between mamba modeling and spatial attention enhancement, making it more adaptive and expressive for medical image segmentation tasks. Next, we provide a detailed explanation of the two core components that constitute the RSSS block: the Vision State-Space Module (VSSM) and the Rceptive-Filed Attention Convolution Module (RFAC).

### Vision state-space module

Inspired by Mamba’s success in maintaining linear computational complexity while also modeling long-range dependencies, as previously mentioned, we constructed the VSSM to extract features from Mamba. As can be seen in Fig 1 (c), we followed the VMamba methodology [25] and chose to process the input features $X\in {\mathbb{R}}^{H\times W\times C}$ through two parallel branches.

In the first branch, we initially expand the channel number of the features through a linear layer to λ times that of the original, i.e., λC, where λ is a predefined channel expansion factor. Then, the features pass through a 3 × 3 depth-wise convolutional layer [46] followed by the SiLU [47] activation function. Subsequently, the output of the SS2D module is normalized by using LayerNorm and combined with the information from the other branch.

In the second branch, the channel number of the features is expanded to λC, using the same technique as in the first branch, followed by processing via the SiLU activation function. Then, the features from the first and second branches are combined by using the Hadamard product to aggregate the information. Lastly, a linear layer projects the channel number back to the original input feature channels *C*, yielding the output *X*_*out*_ with the same shape as the input. This process can be represented by the following equation:

$$\left\{ \begin{array}{l} X_{1}=\text{LN}(SS2D(\text{SiLU}(\text{DWConv}(\text{Linear}(X)))))\\ X_{2}=\text{SiLU}(\text{Linear}(X)),\\ X_{out}=\text{Linear}(X_{1}⊙X_{2}) \end{array}\right.$$ (7)

### 2D selective scan module

**Fig 2:**
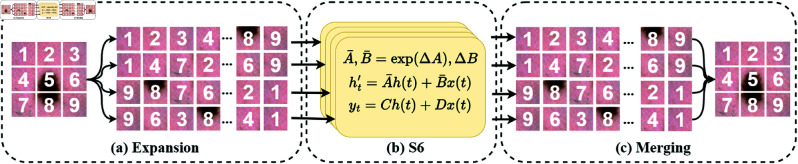
SS2D module. (**a**) SS2D expansion operation; (**b**) Core component of Mamba (S6); (**c**) SS2D merging operation.

The original Mamba is a unidirectional model that can only process 1D information and is thus substantially limited in handling 2D image information. To adapt Mamba for visual tasks and better utilize 2D spatial information, we chose to apply the SS2D module from VMamba as the foundational component of the VSSM, as shown in Fig 2.

Specifically, the SS2D module obtains image patches by unfolding the 2D image in four directions (i.e., from top-left to bottom-right, bottom-right to top-left, top-right to bottom-left, and bottom-left to top-right). These patches are then processed into four different 1D sequences, each of which is processed by an SSM (S6). Lastly, the sequences from different directions are merged to reestablish a complete 2D feature map. Given the input feature $z\in {\mathbb{R}}^{H\times W\times C}$, SS2D processing can be represented by the following equation:

$$\left\{ \begin{array}{l} z_{v}=Expand(z,v),\\ {\bar{z}}_{v}=S6(z_{v}),\\ \bar{z}=Merge({\bar{z}}_{1},{\bar{z}}_{2},{\bar{z}}_{3},{\bar{z}}_{4}), \end{array}\right.$$ (8)

where v∈V={1,2,3,4} represents the four different scanning directions. *Expand*, *S6* and *Merge* denote the expansion, scanning and merging processes, respectively. The S6 module captures long-range information for each sequence according to Eqs. 1 and 2, $\bar{z}$ represents the output feature map of the SS2D module. For more details regarding S6, refer to [25].

### RFAC module

Existing standard convolution operations extract features by performing sliding window operations of convolution. However, there is often overlap between sliding windows, and the parameters of the convolutional kernel are the same within each sliding window. Therefore, the convolution operation cannot capture the position-related information discrepancies in the image, which constrains the effects of the convolution operation. Receptive-field features are represented by the feature maps obtained after the transformation of the original feature map, and these maps comprise non-overlapping sliding windows. The spatial attention mechanism can learn the importance of features in different positions through the attention map. Therefore, the effective combination of the spatial attention mechanism and convolution operation overcomes the limitations of standard convolution operations and paves the way for further development of spatial attention.

**Fig 3:**
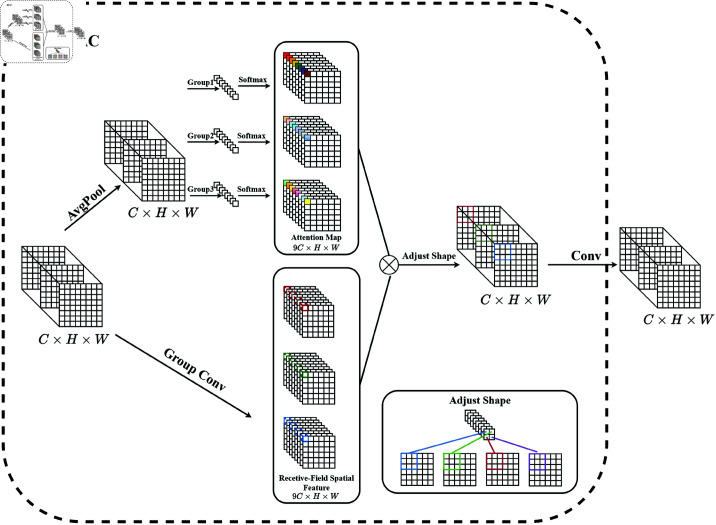
RFAC module, which dynamically determines the importance of each feature in the receptive field.

RFAC utilizes non-overlapping windows within the receptive field to extract features, as shown in Fig 3. RFAC involves multiplying the attention map by the transformed receptive-field features. Specifically, the attention map first applies average pooling (AvgPool) to aggregate the global information of features within each receptive field, performs a 1 × 1 convolution to obtain interaction information, and, lastly, applies the softmax function to emphasize the importance of each feature within the receptive field. The transformed receptive-field features are processed by applying a fast group convolution (Group Conv) operation, which efficiently performs grouped convolutions by splitting channels into groups and processing them in parallel. RFAC can be represented by the following equation:

$$\text{F}=A_{r}\times F_{r}\;=Softmax(g^{1\times 1}(AvgPool(X)))\times ReLU(Norm(g^{k\times k}(X))),$$ (9)

where *A*_*r*_ represents the attention map and *F*_*r*_ represents the transformed receptive-field features obtained via the Group Conv operation. $g^{i\times i}$ indicates a group convolution with kernel size *i* × *i*, *k* indicates the size of the convolutional kernel, and Norm refers to the batch normalization process. Therefore, RFAC can generate a corresponding attention map for each receptive field, which is then multiplied by the convolutional features. This eliminates the insensitivity of standard convolution operations to positional changes within the receptive field, thereby allowing the capture of different features at different positions in the image and realization of positional information awareness.

### Loss function

The mask images used in medical image segmentation are binary or multi-class, medical image segmentation can be viewed as a pixel-level classification task. Therefore, we chose to apply binary cross-entropy (Bce) loss, Dice loss, and cross-entropy (Ce) loss as the loss functions for different tasks, as shown in Eq 10. For the Synapse dataset, we applied Dice loss and Ce loss to form the CeDice loss function and extend their applicability to multi-class problems, as shown in Eq 11. For the ISIC dataset, we applied Dice loss and Bce loss to form the BceDice loss function, as shown in Eq 12.

$$\left\{ \begin{array}{l} L_{Bce}=\frac{1}{N}\sum_{i=1}^{N}\left[y_{i}\log({\hat{y}}_{i})+(1-y_{i})\log(1-{\hat{y}}_{i})\right]\\ L_{Ce}=-\frac{1}{N}\sum_{i=1}^{N}\sum_{c=1}^{C}y_{i,c}\log({\hat{y}}_{i,c}),\\ L_{Dice}=1-\frac{2\left|X\cap Y\right|}{\left|X\right|+\left|Y\right|}, \end{array}\right.$$ (10)

$$L_{CeDice}=\lambda_{1}L_{Ce}+\lambda_{2}L_{Dice},$$ (11)

$$L_{BceDice}=\lambda_{1}L_{Bce}+\lambda_{2}L_{Dice},$$ (12)

where *N* represents the total number of samples and *C* represents the number of classes. *y*_*i*_ and ${\hat{y}}_{i}$ represent the ground truth and predicted values, respectively. *y*_*i*,*c*_ indicates whether the *i*-th sample belongs to the class *c*. If the sample *i* belongs to the class *c*, the corresponding value is 1; otherwise, it is 0. ${\hat{y}}_{i,c}$ is the model’s predicted probability that a sample *i* belongs to the class *c*. |X| and |Y| denote the ground truth and predicted values, respectively. $\lambda_{1}$ and $\lambda_{2}$ are the weight parameters of the loss functions, which were initially set to 1 (see [28] for details).

## Experimental results

### Implementation details

All experiments with our proposed SA-UMamba were conducted in an environment with Ubuntu 22.04, Python 3.8, CUDA 12.2, PyTorch 2.1.0, and two RTX 3090 GPUs. Following previous works, we used image sizes of 256×256 pixels for the ISIC17, ISIC18 and CVC-ClinicDB datasets, and 224×224 pixels for the Synapse dataset. Data augmentation techniques, including random flipping and random rotation, were employed to prevent overfitting. We used the BceDice loss function for the ISIC17, ISIC18 and CVC-ClinicDB datasets and the CeDice loss function for the Synapse dataset. The batch size was set to 32, and the AdamW [48] optimizer was applied. The initial learning rate was set to 10^−3^, with a minimum learning rate of 10^−5^, and the CosineAnnealingLR scheduler [49] was employed. The maximum number of iterations was set to 50, and the training epoch size was set to 300. For pre-trained weights, we used the weights of VMamba-S [25] pre-trained on ImageNet-1k to initialize the parameters of the encoder and decoder in our model.

### Applied datasets

In this study, testing involved the utilization of three datasets commonly used in the medical segmentation field: the Synapse multi-organ segmentation dataset (Synapse), International Skin Imaging Collaboration dataset 2017 (ISIC17), International Skin Imaging Collaboration dataset 2018 (ISIC18) and Colorectal Cancer-Clinic Dataset (CVC-ClinicDB).

The Synapse dataset [50] is a publicly available multi-organ segmentation dataset comprising data on eight types of abdominal organs (aorta, gallbladder, left kidney, right kidney, liver, pancreas, spleen, and stomach). It includes 30 abdominal CT cases, comprising 3779 axial CT images. Following previous studies, we applied 18 cases as the training set and 12 cases as the test set. For this dataset, we used the DSC and 95% HD95 as evaluation metrics for the model.

The ISIC17 and ISIC18 datasets [51, 52] are skin lesion segmentation datasets released by the International Skin Imaging Collaboration (ISIC) in 2017 and 2018. ISIC17 contains 2150 images with segmentation mask labels, and ISIC18 contains 2694 images with segmentation mask labels. Following previous studies [28, 45], we split the datasets into training and testing sets with a 7:3 ratio. For these two datasets, we conducted detailed analyses by using several evaluation metrics, i.e., the mean Intersection over Union (mIoU), DSC, accuracy (Acc), specificity (Spe), and sensitivity (Sen).

The CVC-ClinicDB dataset [53] is the training dataset for the Colonoscopy Polyp Detection Challenge in the MICCAI 2015 competition. It is primarily used for the early detection and diagnosis of colorectal cancer and the dataset consists of 612 high-resolution colonoscopy images. In our work, we follow the approach used in BRAU-Net++ [45] and randomly split the dataset into 490 images for training, 61 images for validation, and 61 images for testing. We evaluated the CVC-ClinicDB dataset using several performance metrics, including the mean Intersection over Union (mIoU), Dice Similarity Coefficient (DSC), accuracy (Acc), precision (Pre) and recall.

### Segmentation results for Synapse dataset

The experimental results show that our SA-UMamba outperformed the CNN- and Transformer-based methods, as well as the latest CNN-Transformer hybrid methods, on both evaluation metrics. It also outperformed our baseline model, VM-UNet, indicating that applying Mamba with spatial attention can result in more efficient modeling. As shown in Table 1, relative to the Transformer-based methods TransUNet [9] and Swin-Unet [10], SA-UMamba significantly increased the DSC by 6.53% and 4.31%, and reduced HD95 by 14.89 and 4.75 mm, respectively. Relative to the Mamba-based VM-UNet method, SA-UMamba increased the DSC by 2.49% and reduced HD95 by 5.57 mm. This demonstrates that Mamba’s global modeling capability is similar to that of Transformers, and that the introduction of spatial attention helps Mamba to more effectively learn spatial semantic information.

**Table 1:** Segmentation results for various methods conducted on Synapse dataset. (The best results are highlighted in bold, and the second best are underlined.)

Methods	DSC(%)↑	HD95(mm)↓	Aorta	Gallbladder	Kidney(L)	Kidney(R)	Liver	Pancreas	Spleen	Stomach
V-Net [37]	68.80	-	75.34	51.87	77.10	80.75	87.84	40.05	80.56	56.98
DARR [54]	69.77	-	74.74	53.77	72.31	73.24	94.08	54.18	89.90	45.96
R50 U-Net [9]	74.68	36.87	87.74	63.66	80.60	78.19	93.74	56.90	85.87	74.16
U-Net [6]	76.85	39.70	89.07	69.72	77.77	68.60	93.43	53.98	86.67	75.58
Attn-UNet [55]	77.77	36.02	**89.55**	68.88	77.98	71.11	93.57	58.04	87.30	75.75
R50 ViT [9]	71.29	32.87	73.73	55.13	75.80	72.20	91.51	45.99	81.99	73.98
TransUNet [9]	77.48	31.69	87.23	63.13	81.87	77.02	94.08	55.86	85.08	75.62
Swin-Unet [10]	79.13	21.55	85.47	66.53	83.28	79.61	94.29	56.58	90.66	79.60
MISSFormer [44]	81.96	18.20	86.99	68.65	85.21	82.00	94.41	**65.67**	**91.92**	80.81
BRAU-Net++ [45]	82.47	19.07	87.95	69.10	**87.13**	81.53	**94.71**	65.17	91.89	**82.26**
VM-UNet [28]	80.53	22.37	86.42	68.27	83.28	80.37	94.65	62.81	89.11	79.36
SA-UMamba (Ours)	**82.54**	**16.80**	88.07	**70.46**	86.46	**83.96**	94.42	65.32	89.89	81.76

Specifically, SA-UMamba outperformed other methods on most organ segmentation tasks, especially the Gallbladder and Kidney (R) images. As shown in Table 1, our SA-UMamba achieved the best DSC of 82.53%. Moreover, compared with the latest CNN-Transformer hybrid method BRAU++ [45], SA-UMamba achieved a lower HD95, demonstrating a level of performance that is comparable to that of the latest Transformer-based methods.

**Fig 4:**
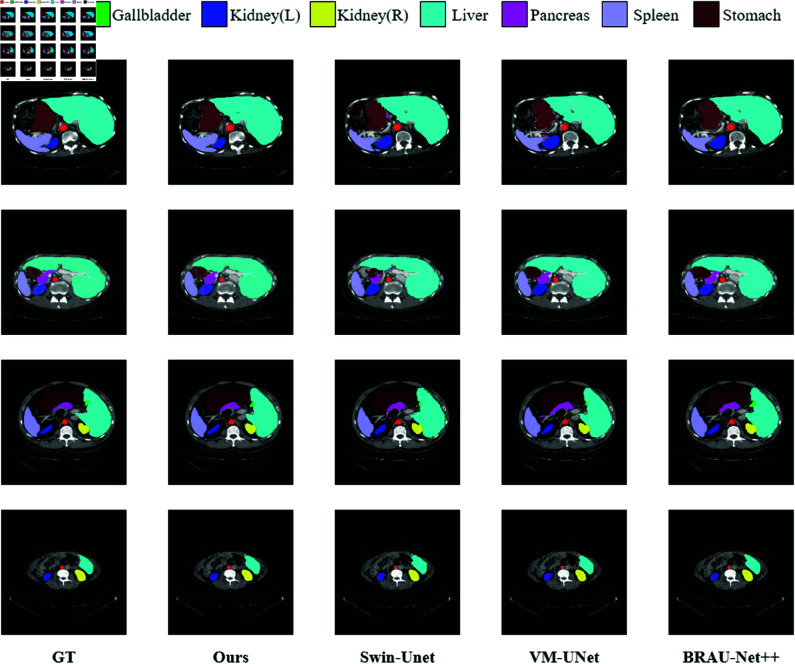
Visualization of segmentation results for different methods on the Synapse dataset.

Fig 4 enables visual analysis of the results for different methods on the Synapse dataset. As shown in Fig 4, our method yielded smoother segmentation results than other methods, as the final images closely resembled the GT images. Even under the condition of small target features, SA-UMamba was able to leverage spatial features to more effectively learn global and long-range semantic information, resulting in superior segmentation performance.

### Segmentation results for ISIC dataset

For the ISIC dataset, we compared SA-UMamba with existing methods. As shown in Table 2, our method outperformed the previous state-of-the-art Mamba model VM-UNet, as well as recent CNN and Transformer models, across various metrics (i.e., mIoU, DSC, and Acc) on the ISIC17 and ISIC18 datasets. Relative to VM-UNet, our proposed SA-UMamba increased by 0.785% on ISIC17 and 0.746% on ISIC18. Although the improvement is not very significant, it demonstrates the effectiveness and robustness of our method on different datasets.

**Table 2:** Segmentation results for various methods conducted on ISIC17 and ISIC18 datasets. (The best results are highlighted in bold, and the second best are underlined.)

Dataset	Model	mIoU(%)↑	DSC(%)↑	Acc(%)↑	Spe(%)↑	Sen(%)↑
	U-Net [6]	76.98	86.99	95.65	97.43	86.82
	UTNetV2 [56]	77.35	87.23	95.84	98.05	84.85
ISIC17	TransFuse [18]	79.21	88.40	96.17	97.98	87.14
	MALUNet [57]	78.78	88.13	96.18	**98.47**	84.78
	VM-UNet [28]	80.20	89.01	96.36	98.03	88.04
	SA-UMamba (Ours)	**80.83**	**89.40**	**96.44**	97.82	**89.60**
	U-Net [6]	77.86	87.55	94.05	96.69	85.86
	UNet++ [32]	78.31	87.83	94.02	95.75	88.65
	Attn-UNet [55]	78.43	87.91	94.13	96.23	87.60
	UTNetV2 [56]	78.97	88.25	94.32	96.48	87.60
ISIC18	TransFuse [18]	80.63	89.27	94.66	95.74	**91.28**
	MALUNet [57]	80.25	89.04	94.62	96.19	89.74
	VM-UNet [28]	80.38	89.12	94.82	**97.29**	87.14
	SA-UMamba (Ours)	8 **0.98**	**89.49**	**94.90**	96.75	89.16

### Segmentation results for CVC-ClinicDB dataset

For the CVC-ClinicDB dataset, we also compare SA-UMamba with existing methods, as shown in Table 3. SA-UMamba outperforms most methods, particularly in DSC (90.41%) and precision (90.59 %), surpassing TransUNet and Attn-Unet. While its recall (86.37%) is slightly lower than Attention U-Net (90.10%), SA-UMamba strikes a strong balance between precision and recall. Compared to the Mamba-based method VM-Unet, our approach improves all performance metrics on the dataset, demonstrating that our work significantly enhances the stability of Mamba-based methods. Overall, SA-UMamba shows superior performance across key metrics, particularly in precision, and proves to be a more robust and promising approach for medical image segmentation.

**Table 3:** Segmentation results for CVC-ClinicDB dataset. (The best results are highlighted in bold, and the second best are underlined.)

Methods	mIoU(%)↑	DSC(%)↑	Acc(%)↑	Pre(%)↑	Recall(%)↑
U-Net [6]	80.91	87.22	98.45	88.24	89.35
Attn-UNet [55]	83.54	89.57	98.64	90.47	90.10
TransUNet [9]	79.95	86.70	98.25	87.63	89.47
Swin-UNet [10]	**84.85**	88.21	**98.72**	90.52	**91.13**
BRAU-Net [58]	77.45	83.64	97.96	84.56	84.20
VM-UNet [28]	80.05	88.6	98.26	89.51	83.29
SA-UMamba (Ours)	82.50	**90.41**	98.61	**90.59**	86.37

### Ablation study

To investigate the impact of various factors on the model performance, we conducted ablation studies using the Synapse dataset. Specifically, we independently evaluated the effectiveness of the pre-trained weights, effectiveness of different RSSS block design choices, and operation of the attention mechanism.

#### Effectiveness of pre-trained weights

Our framework was built based on that of VMamba, which incorporates pre-trained model weights of three different sizes. To explore the impact of different VMamba pre-trained weights on SA-UMamba, we conducted an ablation study under four configurations, i.e., under the conditions of training the model from scratch and using three different scales of pre-trained VMamba weights. The model without pre-trained weights is referred to as Original, whereas those with pre-trained weights of three different sizes are referred to as Tiny, Base, and Small, respectively.

Table 4 details the experimental results for different pre-trained weights on the Synapse dataset. As shown in Table 4, using pre-trained models led to more favorable results than when pre-trained models are not used. Specifically, the Small model demonstrated significant improvement over Original, with the DSC increasing by 5.48% and HD95 decreasing by 28.87%; particularly, it achieved the best DSC and HD95. However, the metrics for the Base model indicated a certain degree of decline relative to the Small model. This indicates that the pre-trained weights significantly influence the performance of the Mamba model. Thus, in the case of SA-UMamba, using the Small-sized pre-trained weights for initialization is optimal.

**Table 4:** Synapse dataset segmentation results for differently scaled pre-trained weights with the same backbone. (The best results are highlighted in bold.)

Methods	DSC(%)↑	HD95(mm)↓
SA-UMamba-Original	78.25	23.62
SA-UMamba-Tiny	80.76	22.50
SA-UMamba-Base	81.20	19.25
SA-UMamba-Small (Ours)	**82.54**	**16.80**

#### Effectiveness of RSSS block design choice

The influence of the RSSS block, as the core module of SA-UMamba, can vary according to its components. Thus, we removed different components within the block to investigate their effects on the model.

In Table 5, baseline represents the model without any additional components. Scale only indicates that using only the residual balancing factor; it does not provide any significant improvement to the model, and may even have a negative impact. RFAC only shows that using only the RFAC module can just lead to moderate improvements in model performance. When both the Scale and RFAC components are used together (Scale+RFAC (Ours)), the framwork demonstrates that combining the balancing factor with RFAC results in significant improvements in model performance, indicating that the optimal Mamba-based medical segmentation model performance requires co-optimization of spatial attention and the Mamba-derived features.

**Table 5:** Synapse dataset segmentation results for different RSSS block design choices. (The best results are highlighted in bold.)

Methods	DSC(%)↑	HD95(mm)↓
Baseline	80.53	22.37
Scale only	77.32	24.74
RFAC only	80.69	19.02
Scale+RFAC (Ours)	**82.54**	**16.80**

#### Operation of the attention mechanism

Attention mechanisms, as a simple and effective way to improve model performance, have been inspired by Transformer-based medical segmentation methods that use different attention calculation mechanisms. Thus, we replaced the RFAC spatial attention with some common spatial and channel attention modules to evaluate the influence of different attention calculation methods on our model. The results are shown in Table 6.

**Table 6:** Segmentation results for different attention calculation methods on the Synapse dataset. (The best results are highlighted in bold.)

Methods	DSC(%)↑	HD95(mm)↓
Baseline	80.53	22.37
Channel Attention [59]	80.93	21.08
CBAM [39]	80.18	22.28
Lightweight CA [40]	80.77	19.39
RFAC [42]	**82.54**	**16.80**

Table 6 shows that channel attention [59] and lightweight CA [40] afforded moderate levels of performance enhancement, whereas CBAM [39] negatively impacted the model performance. Therefore, choosing an appropriate attention calculation method is crucial. In the case of Mamba-based medical segmentation tasks, we believe that RFAC [42] not only overcomes the CNN’s problem of locality, it also emphasizes the feature differences within each receptive field by using adjustment factors to co-optimize the spatial attention and Mamba-derived features. Consequently, RFAC significantly contributes to enhance Mamba performance.

#### Encoder-decoder architectures

Given that the number of RSSS blocks in both the encoder and decoder can significantly influence model performance, we conducted an ablation study to evaluate how variations in the number of these blocks affect the model’s effectiveness. The results are shown in Table 7.

**Table 7:** Ablation study on the number of RSSS blocks in encoder and decoder. (The best results are highlighted in bold.)

Encoder–Decoder	Params(M)↓	FLOPs(G)↓	DSC(%)↑	HD95(mm)↓
{2,2,2,2}–{2,2,2,1}	**43.45**	**7.02**	**82.54**	**16.80**
{2,2,2,2}–{2,2,2,2}	43.60	7.27	81.98	19.02
{2,2,9,2}–{2,2,2,1}	61.46	10.11	81.74	20.60
{2,2,9,2}–{2,9,2,2}	69.94	12.95	80.64	19.02

As shown in Table 7, increasing the number of RSSS blocks in both encoder and decoder leads to a rise in model complexity, resulting in higher parameter count and computational load. However, with the increase of model complexity, the segmentation performance has declined. For example, when the number of blocks increases from {2,2,2,2}–{2,2,2,1} to {2,2,9,2}–{2,2,2,1}, the DSC value decreases from 82.54% to 81.74%, and HD95 value increases from 16.80 mm to 20.60 mm. This indicates that the increased complexity does not yield corresponding improvements in performance and may even negatively affect the model’s generalization ability. Additionally, we found a clear performance advantage of the asymmetric structure over the symmetric one. Based on these findings, we designed SA-UMamba with an asymmetric structure, specifically, {2,2,2,2}–{2,2,2,1} RSSS blocks were assigned to the encoder and the decoder.

#### Comparison of Computational Costs with SOTA methods

To further validate the effectiveness of our proposed method, we compare it with several SOTA models in terms of parameter count and computational complexity, as summarized in Table 8. Although our model exhibits a higher number of parameters compared to conventional convolutional method U-Net and Transformer-based model Swin-Unet, it achieves significantly lower FLOPs while attaining superior segmentation performance in both DSC and HD95 metrics. When compared with VM-UNet, a pure Mamba-based model, our method introduces slightly more parameters and computational cost due to the integration of convolutional operations. However, this design enhances feature extraction capability and contributes to achieving the best overall performance. Furthermore, compared with BRAU-Net++, which combines convolution and Transformer modules, our model reduces the computational burden to nearly half while delivering better DSC. These results demonstrate that, by leveraging the high efficiency of the Mamba architecture along with architectural design, our method achieves a favorable trade-off between performance and computational cost.

**Table 8:** Comparison of model Params(M), FLOPs(G). These test results were obtained on a single RTX3090. (The best results are highlighted in bold.)

Method	Type	Params(M)↓	FLOPs(G)↓	DSC(%)↑	HD95(mm)↓
U-Net [6]	CNN	**7.82**	10.78	76.85	39.70
Swin-Unet [10]	Transformer	27.17	7.72	79.13	21.55
BRAU-Net++ [58]	CNN+Transfomer	50.76	22.27	82.47	19.07
VM-UNet [28]	Mamba	27.43	**4.11**	80.53	22.37
SA-UMamba (Ours)	CNN+Mamba	43.45	7.02	**82.54**	**16.80**

### Discussion

In this work, we propose SA-UMamba, which integrates the global sequence modeling capabilities of Mamba with the local feature extraction strengths of convolutional layers to improve medical image segmentation performance. Although our method outperforms baseline models on most evaluation metrics, it performs slightly worse on a few metrics. We attribute this to specific artifacts in the datasets, such as hair occlusion in ISIC18, which may hinder the extraction of reliable local features and reduce the overall representation quality.

To address this, our future work will explore pure Mamba architectures with inherent global-local modeling capabilities, aiming to eliminate reliance on additional modules and further enhance model generalization. We also plan to reduce computational overhead by designing lightweight Mamba variants that avoid multi-directional scanning. Moreover, we will extend SA-UMamba to a broader range of medical imaging tasks, such as detection and registration, to evaluate its versatility and practical applicability.

## Conclusions

In this paper, we have introduced SA-UMamba, a Mamba-based U-shaped medical segmentation model with spatial attention. We utilized RSSS blocks to construct the encoder and decoder, employing learnable scaling factors to co-optimize Mamba-derived and spatial features. The results of extensive experiments confirmed that our SA-UMamba model outperforms previous Mamba-based medical models. In the future, we will explore optimization strategies for the Mamba model to reduce the number of parameters and further decrease the computational resource requirements, thereby extending the applicability of SA-UMamba.
